# Dopamine, Distraction, and Disruption: Perspectives on How Technology and Generation Z Are Reshaping Medical Education

**DOI:** 10.2196/80791

**Published:** 2026-03-18

**Authors:** Bruno B Andrade, Mariana Araújo Pereira, Katia Avena

**Affiliations:** 1 Laboratório de Pesquisa Clínica e Translacional, Fundação Oswaldo Cruz, Rua Waldemar Falcão, Salvador, Brazil, 55 3176-2202; 2 Instituto de Pesquisa Clínica e Translacional (IPCT), Medicina Zarns, Salvador, Brazil

**Keywords:** medical education, undergraduate medical education, generational differences, generation divide, digital natives, dopamine, Generation Z

## Abstract

This viewpoint reflects on how Generation Z (born between 1995 and 2009), shaped by constant digital engagement, a growing awareness of mental health, and a dopamine-driven environment, is transforming medical education and practice. We explore, from a reflective and interdisciplinary perspective, how the defining characteristics of Generation Z, such as their familiarity with technology, demand for emotional safety, and resistance to traditional hierarchies, might reshape the ways we teach, learn, and practice medicine. Drawing on neuroscience, psychology, sociology, and the medical education literature, this viewpoint emphasizes the need to move beyond knowledge transmission and foster self-regulation, critical thinking, and ethical judgment. We call for a deliberate and compassionate adaptation of medical education to cultivate the skills required for a profession increasingly practiced in a context of overstimulation and complexity.

## Introduction: From Generational Transition to Digital Learning Design

### Background

Medicine has always been a mirror held up to society. Its rhythms, hierarchies, and even its blind spots are shaped by the values, anxieties, and aspirations of those who practice it [1]. Each generation of physicians leaves behind discoveries and treatments, in addition to cultural imprints, unique ways of thinking, diagnosing, and caring. The Hippocratic humility of the ancients, the anatomical fervor of the Renaissance, the clinical detachment of the 20th century, and the evidence-based revolution of the 2000s each emerged from broader generational shifts in how the world is understood.

These generational traits shape medical practice and interact with the challenges of their time. Social, economic, and technological transformations simultaneously affect both the epidemiological profile of populations and how professionals position themselves in relation to care [2]. How a generation understands the body, disease, and the physician-patient relationship is closely linked to the historical conditions in which it operates [3]. Thus, understanding generational tensions and the contexts that produce them is essential for interpreting the current dilemmas faced by educational institutions and health systems.

We are currently living through rapid demographic and epidemiological transitions [4] that demand constant adaptation from educational institutions. However, beyond these challenges, a new factor deserves attention: the arrival of digital natives in medical training. Raised in environments of high digital exposure, frequent stimulation, digital saturation, and dopamine overload, these students, members of Generation Z (Gen Z), may significantly influence both medical education and practice, bringing potential benefits as well as challenges [5,6]. This process has implications that go beyond classrooms and clinics, with possible repercussions for widely recognized professional values in medicine, such as empathy, ethics, and self-control.

This historical context and the challenges of our time underline the need to reflect on the impact new generations may have on how medicine is taught, learned, and practiced. The entry of Gen Z (born between 1995 and 2009) [6-8] into medical education is not merely a generational renewal; it is a cultural, cognitive, and technological transition that challenges traditional models of training and professional conduct [9].

Understanding this transition is crucial for anticipating trends, adjusting curricula, and rethinking the core values of the medical profession, including the need to redefine the competencies expected of future physicians in light of emerging technologies and societal changes [10]. Against this backdrop, this critical viewpoint explores how the characteristics of Gen Z may influence medical education and practice, both in promising and challenging ways. We take digital learning design as our guiding thread and discuss how the neurocognitive and sociocultural characteristics of Gen Z translate into specific demands for medical education.

This viewpoint does not aim to present quantitative data or causal generalizations but rather to reflect on emerging trends in medical education, considering social, cultural, and technological transformations. To this end, it adopts an interpretive approach based on a narrative review of recent literature, published institutional reports, and teaching experiences in different academic contexts. Observations on aspects such as punctuality, emotional regulation, and resilience are used as illustrative examples of formative tensions rather than as population-level estimates. Whenever possible, these interpretations are anchored in peer-reviewed studies; when anecdotal evidence or local reports are used, their limited scope is made explicit. The purpose, therefore, is to generate hypotheses and offer pedagogical directions for future inquiry, situating the debate within a reflective and interdisciplinary perspective.

### Who Is Gen Z? Between Constant Stimulation and Digital Learning Design

According to generational cohort theory, individuals can be grouped by similar behaviors, values, and perceptions shaped by shared life experiences and historical events. Today, 6 living generations are typically recognized: the Silent Generation (or “traditionalists”; born between 1925 and 1945), baby boomers (1946 and 1964), Generation X (1965 and 1979), Generation Y (or millennials; 1980 and 1994), Gen Z (1995 and 2009), and Generation Alpha (2010 and 2024) [6-8,11].

Gen Z now represents a significant portion of the global population, accounting for around 22% of people worldwide and already 27% of the global workforce [11]. This generation grew up amid profound digital, economic, and social transformations [10]. Projections suggest that over their lifetime, they will hold an average of 18 jobs, switch between 6 different careers, and live in approximately 15 different residences, pointing to a reality defined by mobility, adaptability, and multiple reinventions in both personal and professional life [11]. These dynamics reinforce the importance of understanding their motivations, expectations, and challenges, which can particularly impact them within health education and training.

Much has been written about the distinctiveness of Gen Z. As the first true digital natives [8,12], they have not simply acquired technology; they were born into its omnipresence. While expanded connectivity brings clear advantages, growing up in a hyperconnected environment has brought its own challenges. Whereas previous generations fought for access to knowledge, mentors, and opportunities, Gen Z’s struggle is for focus, stillness, and meaning.

*Dopamine Nation* [13] gives voice to this shift. In a world engineered for the overconsumption of pleasure, the brain’s reward system becomes dysregulated. Pleasure leads to pain. Novelty dulls attention. Meaning drowns in noise. This is not simply a sociological curiosity; it is a neurobiological condition [13,14]. In medical practice, such overstimulation can impair core skills like active listening, progressive clinical reasoning, and tolerance for uncertainty, all fundamental to ethical and effective care [15].

At first glance, the traits of this generation appear promising. Gen Z is radically open about mental health, deeply collaborative, socially engaged, and skeptical of inherited hierarchies [14]. They seek to heal, but on their own terms. They reject the stoic pride that previous generations placed on sleep deprivation, emotional suppression, and boundless sacrifice. They speak, with surprising fluency, the language of burnout, boundaries, and balance [16]. This translates into an explicit search for training environments that value emotional safety, horizontality in relationships, and integration between personal and professional life, especially in the face of rapid digital transformation and intergenerational gaps in the use and understanding of technology [11,13,14]. These tensions are not mere contradictions but dispositions that coexist within the same habitus, reflecting the social and institutional pressures that shape the formative experience [17].

Considering this profile, medical education is already feeling the pressure. The old passive learning model—lecture-heavy, memorization-focused, and obedience-based [18,19]—has proven to be less aligned with current preferences. Evidence suggests that Gen Z students tend to prefer more interactive and flexible methods. This generation seeks connection, not coercion [12,20]. This reorientation of expectations resonates with current proposals for medical education reform, which advocate for personalized learning pathways, technological fluency, and humanistic training to prepare students for dynamic health care systems [10].

However, such a shift in expectations also brings with it inherent tensions, particularly when the culture of immediacy collides with the slow, effortful nature of learning, especially within the clinical training required for medical mastery. A generation conditioned by instant feedback and low friction may resist the slow, iterative, and often painful path to mastery [21]. Medical training involves prolonged processes of competence acquisition and, as a rule, does not provide instant gratification. The depth of clinical judgment, the nuance of human physiology, and the texture of uncertainty do not reveal themselves to the impatient or easily bored [22].

These characteristics, particularly strong digital exposure, the need for predictability, and the valuing of rapid returns, directly impact how digital learning experiences should be organized. In practice, this means offering short and clearly delimited units, making objectives explicit, signaling content complexity, providing support to manage difficulties, and ensuring formative feedback that preserves conceptual depth without fostering dependence on immediate answers.

## Redefining the Clinic: Human Connection and Digital Overidentification

We recognize that generational categories are useful as a starting point, but they also have limitations when taken too rigidly. For this reason, we combine the cohort perspective with contributions that broaden the understanding of how social and historical contexts shape educational trajectories. The notion of generational consciousness and distinct groups within the same generation helps situate collective experiences in their time [23].

The concept of habitus, associated with forms of capital and social fields, highlights how dispositions and expectations vary according to cultural and institutional conditions [24]. The idea of the risk society further illustrates how global uncertainties and pressures influence professional choices and educational patterns [25]. Similarly, the humanities and critical social sciences call attention to the limits of approaches centered exclusively on biology. Analyses of neurochemical identities [26], the notion of neuroplasticity as also social [27], and the role of the brain in contemporary definitions of identity [28] reinforce the importance of integrating sociocultural dimensions into neurobiological explanations. Thus, we use both generational and neuroscientific perspectives as complementary lenses, always in dialogue with the social, cultural, and institutional contexts that shape medical education.

These evolving expectations around training and wellness inevitably spill over into clinical environments, where differing generational views on professionalism begin to reshape physician-patient and teacher-student relationships [3,19,21]. Age can influence whether an individual considers a particular behavior professional or unprofessional in clinical settings [19]. The values and traits of each generation may shape how they define and perceive medical professionalism [19,21].

Within this context, the clinic will also shift under the influence of this new generation. Gen Z physicians may stand out in their ability to forge human connections, especially with patients whose struggles mirror their own: anxiety, substance use, technology-related burnout, and identity crises [22]. They may redefine the physician-patient relationship by flattening hierarchies, enhancing transparency, and more seamlessly integrating mental and physical care.

However, this approach risks collapsing professional distance, blurring the line between empathy and overidentification. There is virtue in vulnerability, but medicine must also remain a space where clarity, composure, and decisiveness prevail, especially in times of crisis [29,30]. Beyond the emphasis on individual virtue, it is useful to recognize empathy as part of the “emotional labor” institutionally required [31] and as a repertoire that can be curricularized, measured, and even commodified in certain arrangements [31]. Feminist perspectives on care remind us that empathic practices are shaped by relations of power, the distribution of responsibilities, and the material conditions of work [32]. This implies that educating for empathy requires attention to the structures that sustain it, rather than treating it solely as a psychological attribute of the individual.

Striking a balance between empathy and firmness is particularly challenging in high-pressure contexts, such as emergency situations or difficult conversations, where the physician’s emotional stability is part of the care process [33,34]. These recommendations resonate with the field of Medical Humanities, which, for decades, has promoted practices of narrative reflection, acceptance of failure, and tolerance of ambiguity as foundations of professionalism and ethical care [35]. These challenges are not limited to patient interactions; they arise before and during the training of physicians themselves. Within classrooms and learning environments, students’ behavior reflects deep tensions between contemporary expectations and the traditional ethos of medicine [24].

Institutional reports describe changes in patterns of punctuality, attendance, and responsiveness to feedback, including warnings about behavior considered unprofessional conduct, low tolerance for criticism, and a growing need to address issues of “etiquette” in clinical and educational settings, ranging from dress codes to cellphone use during clinical rounds [21,30].

Among many Gen Z medical students, failure is not merely avoided; it is seen as unacceptable. Anything short of perfection is perceived as invalid. The demand for immediate results and flawless performance reflects habits shaped by short-form content and frictionless interfaces [36]. If a learning experience is not fast, intuitive, or immediately rewarding, it risks being dismissed altogether. By repositioning “failure” as a pedagogical component, this approach aligns with the “medical humanities” literature, which understands it as a resource for learning, professional identity, and patient safety. However, this outlook undermines the iterative, uncertain, and often uncomfortable process required for meaningful learning, not only in medicine but in any complex skill.

In an age of accelerated media and superficial metrics of influence, the appearance of knowledge can overshadow the substance of learning. Popularity, reach, and digital fluency may take precedence over reflective thinking and epistemic humility. These episodes reveal a disconnect between Gen Z’s aspirations for autonomy and well-being and the historical values, norms of professional decorum, and aphorisms that have shaped medical practice for millennia [21,30,37].

Among the most critical consequences of this disconnect is the progressive decline in empathy throughout medical training [38-40], a trend that may be intensified by the psychological distress and emotional exhaustion experienced by many Gen Z students [11,40]. Preserving empathy requires more than idealism; it demands intentionality [41]. Empathy can be cultivated through curricular interventions that promote deep listening, perspective-taking, and the strengthening of interpersonal communication—skills directly linked to clinical performance and patient satisfaction [21,39,41]. Given the risk of empathic decline, especially in emotionally and institutionally stressful contexts common to Gen Z, medical schools must foster psychologically supportive environments and pedagogical practices grounded in structured reflection [40]. Cultivating the affective domain, therefore, requires more than attitudinal content; it calls for safe spaces that enable emotional insight, interpersonal development, and the reinforcement of ethical, compassionate care [11,21].

In medical training, the cognitive and affective domains move in tandem. How a student feels about the world profoundly shapes how they interpret and act within it. The construction of clinical reasoning depends on logic and on the emotional regulation that sustains listening, attention, and discernment amid uncertainty [34]. Dopamine dysregulation is not limited to the patient population; it may also shape physicians themselves. In a profession that demands presence, endurance, and long-term thinking, constant digital stimuli and fragmented attention can erode clinical focus [42].

Although Gen Z is highly familiar with digital technologies and multitasking, these qualities present substantial challenges in medical education [13,41]. The temptations to turn to phones, multitask, or escape discomfort are habits that may compromise patient safety by increasing the risk of missed clinical signs, rushed interpretations, and shallow thinking. Many students in this generation struggle to distinguish fact from opinion [43], weakening their ability to develop critical thinking and undermining clinical reasoning [13,43]. Gen Z is marked by paradox: idealistic yet anxious, collaborative yet individualistic, engaged yet frequently overwhelmed [13]. They often feel lost among an overload of data and information and require active guidance to identify reliable sources and interpret reality more reflectively [13,43].

Medical education must begin to address this not as a moral failing, but as a neurological and cultural condition. Training must extend beyond foundational biomedical knowledge to include practices that promote self-regulation, attention hygiene, and recovery of cognitive depth [21]. This approach is not a luxury; it is now a clinical imperative. This involves, for example, incorporating practices such as mindfulness, self-care strategies, and digital education into the curriculum, preparing students to recognize and manage their own distraction triggers [44]. Embedding structured reflection and self-assessment within digital learning paths can also mitigate cognitive biases and strengthen essential diagnostic skills, such as hypothesis generation and decision-making under uncertainty [43].

The same factors that influence empathy, communication, and professionalism in face-to-face clinical settings also emerge in digital learning environments, whether in the conduct of simulated consultations, the appropriate use of mobile devices, or the handling of sensitive content in online modules. For this reason, e-learning and simulation experiences need to include clear guidance on professional conduct, opportunities for emotional preparation, and spaces for structured reflection.

## Teaching Medicine in the Age of Overstimulation: Technology and Design

### Overview

Medical education has undergone several significant transformations over time. The first of these occurred in the early 20th century, when a robust, science-based curriculum was created that helped establish the medical profession [18,19]. Then, in the second half of the 20th century, innovations such as problem-based learning emerged, aiming to better connect theoretical knowledge to clinical practice [18]. Nowadays, we are experiencing a third major transformation, driven both by a new generation of university students and the ever-evolving needs of health systems.

This reform should not be limited to expanding content or adopting new teaching methodologies. Instead, it requires a comprehensive shift in the definition of core competencies, integrating interdisciplinary knowledge, leadership development, and digital literacy from the undergraduate level [10]. It must focus on developing critical, flexible leaders who are capable of innovating and addressing the complex challenges of modern health care [6]. In line with medical humanities, curricula that normalize formative error, cultivate reflection, and train tolerance for ambiguity tend to sustain attention, empathy, and clinical judgment in contexts of high complexity.

Addressing the challenges of Gen Z requires embedding digital innovation into curricular design. Adaptive learning platforms and learning analytics can personalize content delivery, helping students cope with cognitive overload by tailoring feedback and pacing to individual progress [36,43,45]. Digital portfolios and e-assessment systems, increasingly integrated into competency-based curricula, encourage structured reflection and longitudinal tracking of competencies, reinforcing professional identity development [46]. Simulation-based environments, ranging from high-fidelity clinical scenarios to virtual and augmented reality, provide safe spaces for repeated practice, error tolerance, and the cultivation of resilience [4,45,47], and can strengthen clinical reasoning when coupled with structured debriefings, though they require consistent pedagogical integration and face barriers of cost and adoption [48]. Furthermore, incorporating design principles such as interactivity, multimodality, and user-centered navigation ensures that digital tools meet Gen Z’s expectations for immediacy and visual engagement [14,36]. When combined with mentorship and psychological support systems, these approaches position technology not as a source of distraction but as a vehicle for meaningful learning and professional growth [49,50].

Technology-mediated learning environments offer concrete opportunities to translate the cultural and psychological traits of Gen Z into educational practice. E-learning platforms and adaptive online modules can provide self-paced learning and continuous formative feedback, helping students manage cognitive overload through tailored content and individualized pacing [43,45]. Virtual patient encounters, increasingly embedded in digital curricula, foster clinical reasoning and diagnostic decision-making in interactive and safe contexts that mirror real-world complexity [51,52]. Simulation-based training, whether through high-fidelity scenarios or virtual and augmented reality, allows for repeated practice, error tolerance, and the cultivation of resilience without compromising patient safety [45]. Artificial intelligence (AI)-assisted learning tools, including intelligent tutoring systems and automated feedback algorithms, further expand the potential for personalized learning pathways and real-time adaptive feedback, supporting self-regulated learning while requiring safeguards against over-reliance [5,14,36,53]. Together, these innovations exemplify how educational design can align with the expectations of Gen Z learners while preparing them for the complexities of modern health care.

In this context, it is crucial to understand who these students are. Today, most medical school entrants worldwide belong to Gen Z, with the majority being 24 years or younger [54,55]. With their digital fluency and strong sense of social urgency, they can act as catalysts for this change. Their emotional intelligence, ethical sensitivity, and resistance to traditional hierarchies make them ideal candidates for a more transformative type of education—one that goes beyond the technical to include leadership, adaptability, and a new way of caring that considers local contexts and leverages global resources [6,13,21,56].

Gen Z prefers quiet learning environments with clearly defined objectives, opportunities for small group mentoring, and greater integration of technology into classrooms [11]. They also demonstrate increased sensitivity to emotionally charged content, including topics related to abuse or death [11,57]. This has driven a growing demand for the use of content warnings [57] and the creation of safe spaces within medical curricula, challenging the long-standing tradition of exposing students to difficult topics without prior emotional preparation [11,57].

In addition, Gen Z’s engagement with health is strongly shaped by technology and social media. They tend to prefer visual content, such as short videos, over traditional texts [13,14,46]. Their expectations emphasize speed, convenience, and the availability of digital interactions [56]. This generation also favors approaches that integrate emotional and physical well-being [14,21].

Another noteworthy shift is their evolving perception of volunteerism. Gen Z tends to reject traditional charity-based models and instead prefers technological and entrepreneurial solutions to social problems [11]. This may reduce participation in conventional student-run clinics, but it creates space for more innovative social impact initiatives to be integrated into the curriculum.

Among the interventions we consider most promising for addressing the identified challenges, three stand out as high priorities: (1) the use of simulation environments with structured debriefing, capable of strengthening clinical reasoning and patient safety [58]; (2) the use of digital portfolios and longitudinal formative feedback, which support the development of professional identity and student self-regulation; and (3) the adoption of adaptive platforms and learning analytics, which help mitigate cognitive overload by adjusting content and pacing to individual needs [36]. Complementarily, practices of attentional hygiene, such as mindfulness and digital literacy, as well as explicit guidelines for professionalism in virtual settings, provide support for the development of self-regulation and the preservation of ethical care. Taken together, these strategies outline a practical path to align technological innovation, cognitive depth, and humanistic formation, responding in an integrated way to the specific demands of Gen Z in medical education.

Beyond generational dispositions, these dynamics are co-produced by institutional choices. Curricular structures, the organization of formative work, and, above all, assessment regimes shape what students prioritize [59]. Systems based on superficial metrics tend to reinforce immediacy and perfectionism, whereas programmatic assessment and entrustable professional activities (EPAs) [60], with frequent low-stakes feedback cycles, promote formative learning and tolerance for error [42]. In addition, the hidden curriculum and organizational culture communicate norms and expectations that can amplify or mitigate these tensions [61], and the quality of feedback cultures depends on institutional design and supervisory practices. Finally, organizational factors are implicated in student well-being and burnout, indicating that interventions must combine individual support with structural change.

### Competency Development in Digital Ecosystems

Medical schools of the future will need to move beyond simply integrating science and care or adopting competency-based curricula [35]. There is a growing demand for individualized learning processes and immersive clinical experiences that prepare students to work in increasingly complex health care systems [17].

Faced with Gen Z’s presence in academic settings, schools must develop new technologies and tools to manage the learning environment, as well as new assessment methods to measure competency acquisition [17]. Many institutions are already responding to these challenges by offering immersive experiences within health systems, allowing students to engage with patient-centered care and collaborative practice [35]. Schools are also implementing educational trajectories based on EPAs [60], using portfolios for guided self-assessment and personalized goal-setting based on performance data [5,62].

Moreover, EPAs can be understood not only as assessment instruments but also as structural interventions capable of mitigating some of the challenges described throughout this article. By clearly defining performance expectations in progressively entrusted clinical activities, EPAs reduce the anxiety related to ambiguity and fear of failure, which are common among Gen Z students [42]. The model of supervised progression, based on levels of trust, provides continuous and calibrated feedback, reconciling this generation’s need for rapid returns with the preservation of formative depth [63]. In addition, the longitudinal nature of EPAs supports the integration of cognitive, affective, and professional competencies, promoting both clinical reasoning and the development of professional identity within digital learning ecosystems [64].

These transformations must also be understood in dialogue with established debates in medical education. The literature on professional identity formation shows that becoming a physician is a gradual process of socialization that involves both ideals and contradictions experienced in daily practice [65,66]. At the same time, some of these tensions are expressed in the so-called hidden curriculum, a set of implicit norms and messages that often reinforce hierarchies and practices that diverge from the formal curriculum [61]. Finally, our argument also aligns with the sociology of medical knowledge, which recognizes medicine as a cultural and historical construction, shaped by regimes of truth and forms of legitimizing knowledge [67].

We are living in a time of unprecedented informational access and, paradoxically, growing uncertainty about what truly matters. For Gen Z, navigating this overload requires more than search skills; it requires the ability to filter, discern, and transform data into clinical wisdom. Medical education must rise to this challenge, fostering competencies that go beyond technical knowledge to embrace critical thinking, adaptability, and ethical judgment [4,12,15,17,18,22,68]. This aligns with the emerging vision of a medical student profile capable of navigating AI-based systems, leading innovations, and promoting global health [20].

We would be wrong to romanticize previous generations. The stoicism of the past often came at the cost of silence, loneliness, and unprocessed trauma. However, we must also avoid the pendulum swing toward fragility. Medicine is hard; it asks of us the ability to delay pleasure, hold ambiguity, and face suffering without paralysis. The literature on dopaminergic dysregulation suggests possible difficulties in these tasks [69]. Nevertheless, the solution is not nostalgia for a lost generation; it is the deliberate cultivation of new skills for a new world.

### Cultivating New Skills for a New World

As this viewpoint has argued, the arrival of Gen Z signals profound changes in medical education, requiring adaptations in curricula, infrastructure, psychological support systems, and pedagogical approaches to meet their unique characteristics.

Perhaps Gen Z, with its hunger for authenticity, can lead us there if we, the educators and leaders, are willing to challenge them gently but firmly. It is recommended to model attention instead of distraction, to provide structured mentorship rather than demand performance, and to promote intellectual discipline alongside emotional openness to dialogue. If we dare to teach them how to be bored again, how to wait, and how to listen deeply—not just to patients, but to themselves.

A generation shaped by constant digital stimulation is entering the most human of professions. The impact of this process will be significant and will depend on how curricula and learning environments are adapted to respond to these generational characteristics. Among the possibilities are educational design strategies that support structured and responsible learning: adaptive modules that adjust the pace of study to individual needs; dashboards that allow students to monitor their progress; electronic portfolios that document acquired competencies; simulation environments, including virtual or augmented reality, that provide safe and repeated practice; AI tools designed to guide learning in a calibrated manner [5,36]; and institutional policies for digital well-being, such as device-free periods or reduced notifications, which help protect attention and reduce cognitive overload.

By integrating evidence on neurocognition and Gen Z preferences with principles of digital design, we propose a framework that combines personalization with depth, efficiency with reflection, and technology with professionalism. The effectiveness of these strategies should be tracked through learning metrics (retention, transfer, and diagnostic reasoning), well-being indicators, and measures of professionalism in digital environments.

## Future Directions: Specific and Testable Questions

### Overview

Building on the interpretive lens of this viewpoint, future research should examine how the generational characteristics of Gen Z interact with curricular structures, assessment systems, and digital learning environments. To advance beyond reflection and provide an empirical foundation for innovation in medical education, we propose the following specific, testable questions:

- Does attentional self-regulation training improve learning and clinical reasoning in Gen Z learners?
- Do adaptive e-learning modules reduce perceived cognitive load for Gen Z students compared to previous cohorts?
- Can structured reflection mitigate the longitudinal decline in empathy or professionalism among Gen Z learners?
- Does AI-assisted tutoring improve diagnostic calibration in Gen Z learners without fostering over-reliance?
- Do portfolios with frequent formative feedback strengthen professional identity and self-regulation among Gen Z learners?
- Do coaching and structured mentoring programs reduce burnout and attrition among Gen Z medical students?

Taken together, these questions outline an empirical agenda that moves beyond broad generalizations about generational traits. They encourage rigorous testing of how technology-mediated interventions, assessment innovations, and institutional supports can shape the learning experiences of Gen Z in medical education, while also offering transferable insights for future cohorts.

### Conclusions

Gen Z is a generation marked by familiarity with technology, a strong emphasis on emotional safety, and lower adherence to traditional hierarchies. Their presence in medical education calls for deliberate adaptations in curricula, student support, and the design of digital learning experiences. By aligning technological interventions with pedagogical principles and consistent assessment strategies, it is possible to combine personalization with depth, fostering sustained attention, critical thinking, and ethical judgment in contexts of high stimulation and complexity.

## Abbreviations

AI: artificial intelligence
EPA: entrustable professional activity
Z: Generation Z
